# Determinants of subnational disparities in antenatal care utilisation: a spatial analysis of demographic and health survey data in Kenya

**DOI:** 10.1186/s12913-020-05531-9

**Published:** 2020-07-18

**Authors:** Kefa G. Wairoto, Noel K. Joseph, Peter M. Macharia, Emelda A. Okiro

**Affiliations:** 1 Population Health Unit, Kenya Medical Research Institute-Wellcome Trust Research Programme, Nairobi, Kenya; 2 Centre for Tropical Medicine and Global Health, Nuffield Department of Clinical Medicine, University of Oxford, Oxford, OX3 7LJ UK

**Keywords:** Antenatal care, Determinants, Mapping, Spatial variation, Sub-national, Kenya

## Abstract

**Background:**

The spatial variation in antenatal care (ANC) utilisation is likely associated with disparities observed in maternal and neonatal deaths. Most maternal deaths are preventable through services offered during ANC; however, estimates of ANC coverage at lower decision-making units (sub-county) is mostly lacking. In this study, we aimed to estimate the coverage of at least four ANC (ANC4) visits at the sub-county level using the 2014 Kenya Demographic and Health Survey (KDHS 2014) and identify factors associated with ANC utilisation in Kenya.

**Methods:**

Data from the KDHS 2014 was used to compute sub-county estimates of ANC4 using small area estimation (SAE) techniques which relied on spatial relatedness to yield precise and reliable estimates at each of the 295 sub-counties. Hierarchical mixed-effect logistic regression was used to identify factors influencing ANC4 utilisation. Sub-county estimates of factors significantly associated with ANC utilisation were produced using SAE techniques and mapped to visualise disparities.

**Results:**

The coverage of ANC4 across sub-counties was heterogeneous, ranging from a low of 17% in Mandera West sub-county to over 77% in Nakuru Town West and Ruiru sub-counties. Thirty-one per cent of the 295 sub-counties had coverage of less than 50%. Maternal education, household wealth, place of delivery, marital status, age at first marriage, and birth order were all associated with ANC utilisation. The areas with low ANC4 utilisation rates corresponded to areas of low socioeconomic status, fewer educated women and a small number of health facility deliveries.

**Conclusion:**

Suboptimal coverage of ANC4 and its heterogeneity at sub-county level calls for urgent, focused and localised approaches to improve access to antenatal care services. Policy formulation and resources allocation should rely on data-driven strategies to guide national and county governments achieve equity in access and utilisation of health interventions.

## Background

Approximately 0.3 million maternal deaths and 2.6 million stillbirths occurred globally in 2015 with sub-Saharan Africa (SSA) accounting for most of these deaths at 66% and 40% respectively [1, 2]. Between 30 to 50% of maternal mortality is due to inadequate care during pregnancy, while two-thirds of stillbirths are antepartum caused by maternal infections and pregnancy complications [3]. These deaths are preventable through services offered during antenatal care (ANC) [3, 4]. ANC visits are aimed at improving triage and timely referral of high-risk women and include educational components and should ideally avert most health complications that may affect the mother or the newborn [4]. Until 2016, the World Health Organization (WHO) recommended at least four ANC visits later revised to eight visits in line with new evidence supporting improved safety during pregnancy through increased frequency of maternal and fetal assessment shown to be associated with a reduced likelihood of perinatal deaths [5, 6].

Countries have routinely monitored the coverage of ANC utilisation and its predictors at national and regional levels through household sample surveys [7]. Typically two ANC coverage indicators are monitored, ANC1 defined as the proportion of women aged 15–49 years who received ANC services provided by a skilled birth attendant (doctor, nurse or midwife) at least once during pregnancy and ANC4 for those who attended four or more visits [8]. In SSA, only 80% of pregnant women accessed ANC1, and only 52% received ANC4 in 2018 [8]. The timing (initiation of first ANC visit) is also monitored and plays a crucial role in determining the completion of the recommended visits.

Tracking coverage at global, regional or country-level is essential for macro-level comparisons. However, analysis at this level obscures significant variations within a country, popularly known as “masking the unfinished health agenda” [9]. The Sustainable Development Goals (SDGs) enshrines health equity based on its fundamental principle of leaving no one behind and with a focus on reaching those who are most marginalised first [10, 11]. Lack of data powered to provide precise and reliable estimates at units of decision-making hinders the description of the subnational heterogeneities [12]. Recent advancement in mapping and statistical techniques have allowed mapping of child survival and its determinants at a fine spatial resolution [13–16]. However, the variation of ANC utilisation and its predictors at lower geographical units of decision making remains imperfectly described in Kenya to facilitate policy formulation and targeted interventions [17].

In the current study, we leverage on small area estimation (SAE) techniques to map ANC4 utilisation at the sub-county level and identify factors affecting ANC utilisation using the data from the Kenya demographic and health survey conducted in 2014 (KDHS 2014).

## Methods

### Country context

**Fig. 1:**
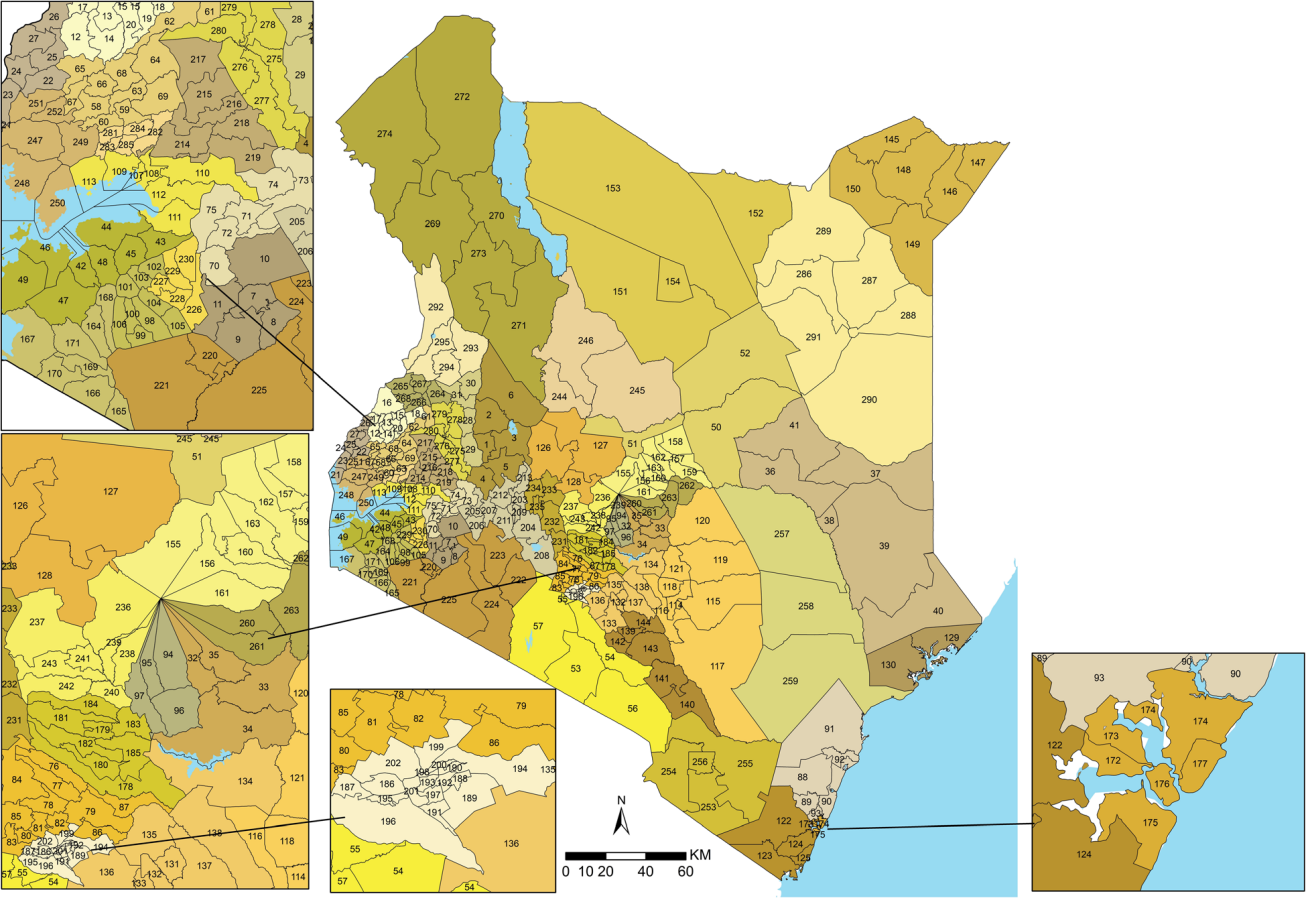
The map of Kenya showing 47 counties (colored) and 295 sub-counties (numbered). The extents of major lakes and the Indian Ocean are shown in light blue. The names of the counties and sub-counties corresponding to the displayed numbers are presented in Additional file 1. Source: author generated map

The Millennium Development Goals (MDGs) era saw Kenya make substantial gains in maternal and newborn health. Following an increase in maternal mortality in the 1990s, the trend was reversed with a 39% reduction in maternal mortality rates (MMR) from 590 per 100,000 live births in 1998 to 362 in 2014 [18]. The units of administration and health planning were revised to 47 counties in 2013 when Kenya adopted a decentralised system of governance (Fig. 1 and additional File 1) [19, 20], and are further divided into 295 sub-counties (Fig. 1 and Additional File 1). Kenya’s health sector is pluralistic with governmental, non-governmental and privately managed health facilities. The structure of service delivery is hierarchical with six tiers, namely community level followed by dispensaries, health centres, primary referral, secondary referral, and tertiary facilities.

There are over 11,000 health facilities in Kenya with about 6000 public health facilities managed by either the ministry of health, local authorities, faith-based organisations and non-governmental organisations capable of offering general health services to the public [21–24]. ANC services are available through these health facilities. Since independence, the government of Kenya has made substantial progress in making healthcare services affordable and accessible to women and children by putting into place different policies affecting access and utilisation [25–34]. Since 2013, all services at government outpatient facilities and maternity services have been offered free of charge [35, 36].

### Data

ANC utilisation, socioeconomic, and demographic data on pregnant women were obtained from KDHS 2014. The survey employed a two-stage sampling design on a national sampling frame of 5360 clusters. One thousand six hundred and twelve (1612) clusters were selected with equal probability, 995 in urban and 617 in rural areas. In the second stage, 40,300 households were selected. Additional data on high-resolution travel time to the nearest health facility were obtained from a study by Alegana et al., 2018 [37]. In brief, travel time to the nearest public health facility was computed based on a cost distance algorithm while factoring in different models of transport and travelling speeds [37]. The method calculates the cumulative travel time associated with travelling from a cluster to the nearest health facility along the shortest possible route. Each DHS cluster was assigned a travel time based on its average time on 2 km (urban) or 5 km (rural) buffer to minimise the random displacement of DHS survey clusters [38–40].

Based on a review of literature assessing the association between ANC use and its determinants [38, 41–46], candidate variables were abstracted from KDHS 2014. They included maternal education, birth order, household wealth, household residence type, marital status, ethnicity, parity, age at first marriage/cohabitation, place of delivery, sex of household head, religion, maternal age and time to the nearest health facility [37].

### Factors associated with ANC4 utilisation

Univariate regression models were used to assess the crude association between each of the determinants and ANC4 utilisation. Variables were included in the multivariate modelling stage if the *p*-value was less than 0.20. Multi-collinearity among predictors was assessed using variance inflation factors (VIF), whereby VIF > 3 indicated highly collinear variables [18]. A hierarchical mixed-effect logistic regression model was used due to the nesting structure and multistage sampling design of the KDHS 2014 [47]. County was included as a random effect to account for region-specific contextual factors (e.g. health financing). Bayesian Information Criteria (BIC) was used to assess the fit of the models using forward variable selection. The models were implemented using “*lme4*” package [48] in R software (version 3·5·2) and StataCorp. 2014 (Stata Statistical Software: Release 14. College Station, TX: StataCorp LP).

### Modelling sub-county coverage using small area estimation

Additional file 2 summarises the analytical processes used to estimate the coverage of ANC4 and its determinants at the sub-county using SAE techniques to smooth both the coverage of ANC4 and significant determinants of ANC utilisation. Individual data on ANC utilisation from KDHS 2014 were collapsed to either 0 (< 4 ANC visits) or 1 (≥ 4 ANC visits). Using Global Positioning System (GPS) cluster coordinates; the individual data was assigned to the respective sub-counties through a spatial join in ArcMap 10.5 (ESRI Inc., Redlands, CA, USA). The weighted number of women who had at least four ANC visits was then computed in StataCorp. 2014 [Stata Statistical Software: Release 14. College Station, TX: StataCorp LP] at sub-county adjusting for the survey sampling design and applying survey weights. A binomial formulation with a logit link function was implemented with a spatial structured random effect (*μ*_*i*_) to account for unmeasured spatial risk factors for ANC use and unstructured random effect (*ν*_*i*_) to account for area-specific characteristics (Eq. 1).

Spatial smoothing of ANC4 and covariates

1 $$ \mathrm{Log}\left\{\frac{p(i)}{1-p(i)}\right\}=\alpha +{\mu}_i+{\nu}_i $$

The spatial dependence (v) was represented through a neighbourhood matrix that defined a set of adjacent neighbours for each sub-county (i) and modelled through a conditional autoregressive (CAR) process. In this formulation, the parameters in one sub-county were influenced by the average of the neighbouring sub-counties. The Besag-York -Molliè (BYM) 2 CAR model [49] was used as it better accounts for identifiability and scaling. Other formulations [50, 51] did not perform any better when tested during model formulation and evaluation. Two sub-counties were defined as neighbours if they shared either a boundary or a node (queen adjacency) because each sub-county had at least an identified neighbour in this definition as opposed to distance-based and rook adjacency (neighbours based on boundary only).

Covariates were not used to assist in modelling ANC4 coverage at the sub-county level to avoid the likelihood of creating a covariate driven metric as opposed to data-driven utilisation rates despite their ability to lower the standard errors [52]. The observed ANC4 utilisation rates were regarded as the result of all possible socioeconomic, demographic and environmental factors that influence ANC trends. Besides, the census of all covariates that would influence ANC4 are neither available, nor are they error-free (unbiased). Thus, the SAE models relied fully on the ANC4 empirical data for the generation of coverage maps. Similar model formulations have been applied elsewhere [12, 14].

The areal level models were run in R software (version 3·5·2) using the R-INLA package. The posterior estimates of ANC4 coverage were then mapped at sub-county level in ArcGIS 10.5 (ESRI Inc., Redlands, CA, USA). Model predictive performance was assessed through cross-validation using a 10% randomly selected hold-out sample and the correlation, root-mean-square-error and the bias computed. The interpretation of the statistics is relative with lower values of root-mean-square error indicating a better fit; a higher correlation suggests an association between the observed and predicted model values and hence preferred [53]. The coverage of the significant variables at sub-county was estimated and mapped using the same framework.

### Ethics approval

This study used secondary data only, which is publicly available to registered users from online data repositories. The procedures and questionnaires for DHS surveys have been reviewed and approved by the ICF International Institutional Review Board (IRB). The ICF International IRB ensures that the survey complies with the U.S. Department of Health and Human Services regulations for the protection of human subjects (45 CFR 46).

## Results

### Participants characteristics

A total of 14,858 women aged between 15 and 49 years had at least one pregnancy each in the five years preceding the KDHS 2014 survey and in theory expected to attend the recommended number of ANC visits during the pregnancy period. Weighted estimates show that 96% of women had at least one ANC visit and 58% had at least four ANC visits. More than 90% of the women in our sample had at least primary school education (90.4%). In contrast, approximately 3 in 5 women (60.4%) came from a household of higher socioeconomic status based on the household wealth index (Table 1). Most women (61.4%) were residing in rural areas in 2014, with a majority being married (81.5%). Almost two-thirds (66.1%) of the deliveries occurred at a health facility with most births occurring at a public health facility (49%). Thirty-five per cent of the women were married before their 18th birthday while 9.6% had at least seven children. Table 1 provides a summary of the characteristics of the study participants.

**Table 1:** Socioeconomic and demographic characteristics of women aged 15–49 who had a live birth in the five years preceding the 2014 Kenya Demographic and Health Survey (*n* = 14,858) and the factors associated with antenatal care utilisation for at least four visits from a bivariate model in Kenya

**Variable**	**Category**	**N** **Weighted Proportion (%)**	**Odds Ratio** **(95% CI)**	***P*-value**
Maternal education	No Education	2739 (9.57)	**Ref**	
	Primary	7813 (54.55)	1.41 (1.17–1.69)	< 0.0001
	Secondary	3200 (26.17)	2.11 (1.67–2.66)	< 0.0001
	Tertiary	1106 (9.72)	5.93 (4.63–7.59)	< 0.0001
Wealth Quintile	Lowest	4461 (20.25)	**Ref**	
	Second	3035 (19.30)	1.35 (1.18–1.54)	< 0.0001
	Middle	2618 (18.43)	1.73 (1.49–2.01)	< 0.0001
	Fourth	2459 (19.26)	2.24 (1.87–2.67)	< 0.0001
	Highest	2285 (22.75)	3.92 (3.13–4.90)	< 0.0001
Religion	Roman catholic	2863 (18.99)	**Ref**	
	Protestant/Other Christian	9439 (71.43)	0.91 (0.79–1.06)	0.233
	Muslim	2143 (7.12)	0.79 (0.62–1.00)	0.05
	No religion	349 (2.25)	0.49 (0.36–0.66)	< 0.0001
	Other religions	39 (0.21)	0.53 (0.26–1.07)	0.078
Residence	Urban	5146 (38.57)	**Ref**	
	Rural	9712 (61.43)	0.60 (0.53–0.69)	< 0.0001
Marital status	Married	12,251 (81.45)	**Ref**	
	Never Married/ Divorced/ Widowed /Separated	2607 (18.55)	0.72 (0.65–0.80)	< 0.0001
Place of delivery	Health Facility	8716 (66.12)	**Ref**	
	Non-health facility	6123 (33.88)	0.42 (0.38–0.47)	< 0.0001
Birth Order	≤4	10,840 (77.29)	**Ref**	
	≥5	4018 (22.71)	0.69 (0.61–0.77)	< 0.0001
Ethnicity	Kalenjin	2234 (13.08)	**Ref**	
	Kikuyu	1959 (18.64)	1.28 (0.97–1.69)	0.083
	Kamba	1261 (10.76)	1.20 (0.91–1.57)	0.196
	Kisii	793 (5.53)	1.02 (0.60–1.73)	0.946
	Luhya	1779 (15.77)	1.00 (0.74–1.37)	0.975
	Luo	1516 (11.68)	1.28 (0.93–1.76)	0.129
	Other tribes	5313 (24.53)	1.13 (0.82–1.55)	0.454
Maternal Age	≤ 24	4305 (29.84)	**Ref**	
	25 - ≤ 34	7273 (49.76)	1.22 (1.07–1.38)	0.002
	> 34	3280 (20.40)	1.15 (0.99–1.34)	0.073
Household Head	Male	10,245 (71.51)	**Ref**	
	Female	4613 (28.49)	0.98 (0.89–1.08)	0.714
Parity	1–3	8891 (65.42)	**Ref**	
	4–6	4219 (25.00)	0.76 (0.69–0.84)	< 0.0001
	≥7	1748 (9.59)	0.58 (0.49–0.68)	< 0.0001
Age at first marriage	< 18	5297 (34.47)	**Ref**	
	≥18	8400 (65.53)	1.37 (1.24–1.51)	< 0.0001
Travel time to nearest Health Facility	< 30	14,124 (97.97)	**Ref**	
	≥30	734 (2.03)	0.55 (0.40–0.75)	< 0.0001

### Subnational coverage of at least four ANC visits

**Fig. 2:**
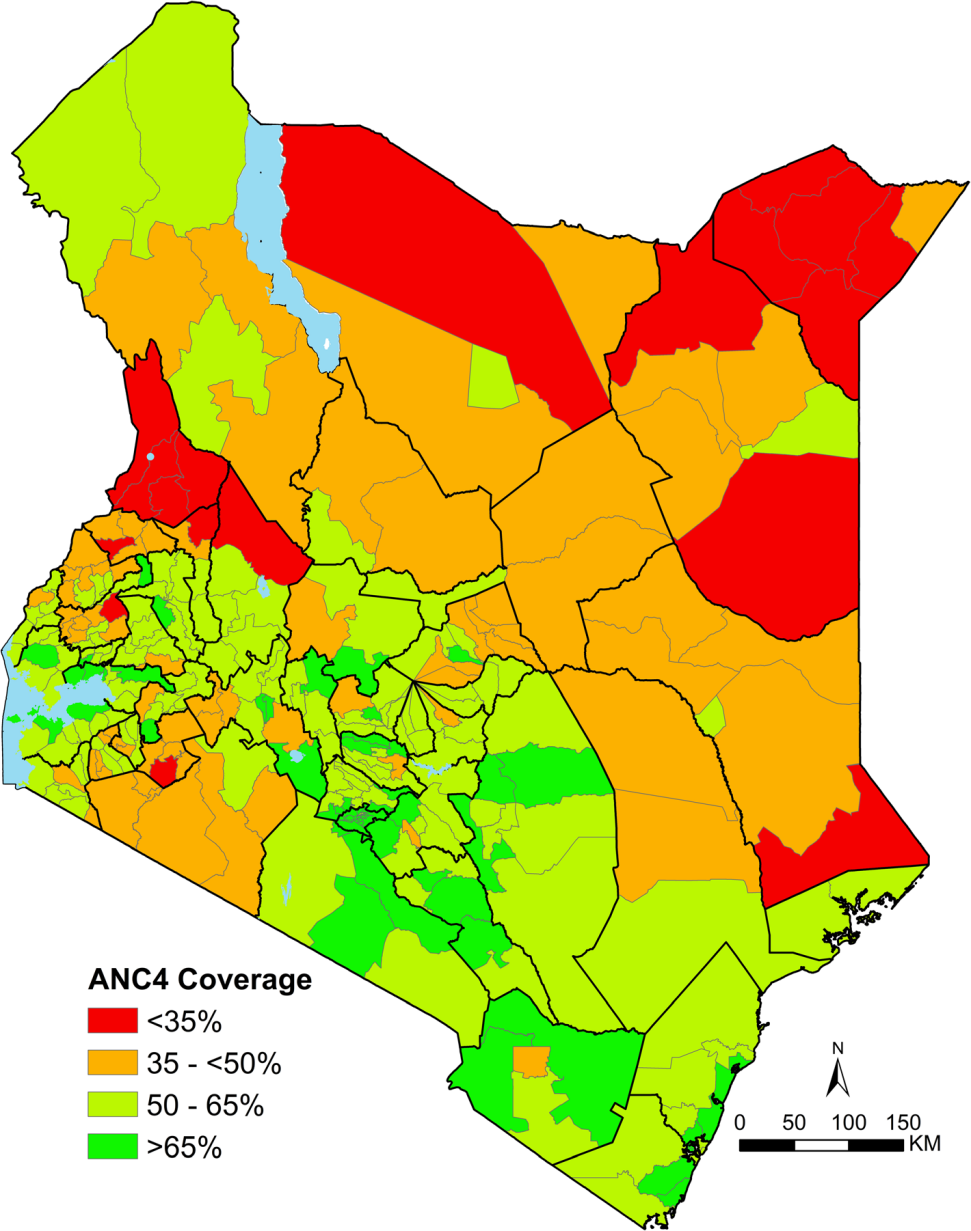
Map showing the coverage of at least 4 ANC visits at sub-county level based on the 2014 Kenya, Demographic and Health Survey. The coverage is classified in four classes ranging from < 35% (red), 35- < 50% (brown), 50–65% (light green) to > 65% (dark green). Source: author generated map

National modelled estimates show that 95.6% [95% CI: 95.2–95.9] of the pregnant women had attended at least one ANC visit in 2014 and three in five had participated in at least 4 ANC visits (57.7% [95% CI: 56.9–58.5]). ANC coverage estimates were computed for all 295 sub-counties. The spatial model had a root mean square error of 13.0, a mean absolute error of 7.2 and a correlation coefficient of 0.813 between the observed and smoothed values. The estimates reveal significant cross-country heterogeneities at the sub-county level (Fig. 2) ranging from 16.9% [95% CI: 9.7–26.8] in Mandera West sub-county to 77.7% [95% CI: 62.5–88.6] in Ruiru sub-county (Fig. 2; Additional file 3).

Sixty-nine percent of the sub-counties (204/295) had a mean coverage of at least 50% of women attending at least four antenatal care visits. These sub-counties were mostly in Central and the South-eastern part of Kenya along the Indian Ocean and some parts of western Kenya along Lake Victoria. Twenty sub-counties had ANC4 attendance of over 70%. They included Kibra, Makadara, Mathare, Roysambu, Dagoretti North (Nairobi county), Nakuru Town West, Naivasha (Nakuru county), Mathioya, Kiharu (Murang’ a county), Changamwe (Mombasa county), Kajiado East, Kajiado North (Kajiado county), Kikuyu, Ruiru (Kiambu county), Muhoroni (Kisumu county), Matungulu (Machakos county), Kibwezi West, Makueni (Makueni county), Msambweni (Kwale county) and Rabai in Kilifi county (Fig. 2). Among the 20 sub-counties, only four sub-counties (Ruiru, Rabai, Makadara and Nakuru Town West) had coverage of over 75% (Fig. 2 and Additional File 3).

Geographically, sub-counties in the Northern and North-Eastern regions had the lowest utilisation of ANC4. A total of 18 sub-counties (6.1%) had a coverage of less than 35% namely Tiaty (Baringo county), Chepalungu (Bomet county), Marakwet East (Elgeyo Marakwet county), Ijara (Garissa county), Malava (Kakamega county), Banissa, Lafey, Mandera North, Mandera South, Mandera West (Mandera county), North Horr (Marsabit county), Saboti (Trans Nzoia county), Wajir North, Wajir South (Wajir county), and North Pokot, Pokot Central, Pokot South, West Pokot, all in West Pokot county (Fig. 2 and Additional File 3).

### Determinants of ANC4 utilisation and their variation sub nationally

Table 1 shows the results of bivariate logistic regression analysis based on 13 candidate variables. Based on the *p*-value, all the factors except sex of the household head were found to have a significant bivariate relationship with ANC coverage and were included in the multivariate analysis (Table 2). The parsimonious model based on BIC had six variables: age at first marriage, place of delivery, maternal education, birth order, marital status and household wealth (Table 2).

**Table 2:** Hierarchical mixed-effects logistic regression model odds ratios of at least four ANC visit among women in the reproductive age (15–49 years) who had at least a live birth, 5 years preceding the 2014 Kenya Demographic and Health Survey

**Covariate**	**Categories**	**Odds Ratio** **(95% CI)**	***P*-value**
**Age at First Marriage/ cohabitation**	**< 18**	**Ref**	
	≥18	1.07 (0.97–1.18)	0.199
**Place of Delivery**	**Health Facility**	**Ref**	
	Non- health Facility	0.54 (0.48–0.61)	< 0.0001
**Maternal Education**	**No Education**	**Ref**	
	Primary	1.07 (0.89–1.30)	0.463
	Secondary	1.33 (1.06–1.67)	0.015
	Tertiary	3.00 (2.29–3.93)	0.0001
**Birth Order**	**≤4**	**Ref**	
	≥5	0.88 (0.78–0.99)	0.027
**Marital Status**	**Married**	**Ref**	
	Never Married/ Divorced/ Widowed /Separated	0.83 (0.75–0.93)	0.001
**Wealth Quintile**	**Lowest**	**Ref**	
	Second	1.19 (1.04–1.36)	0.013
	Middle	1.34 (1.14–1.58)	< 0.0001
	Fourth	1.45 (1.22–1.72)	< 0.0001
	Highest	2.06 (1.60–2.65)	< 0.0001
**Random Effect**		**Variance**	**Std. error**
**County**		0.20 (0.11–0.36)	0.0611
**Intra class correlation coefficient (ICC)**	0.09 (0.06–0.13)		

Probability of ANC4 utilisation increased across wealth quintiles; the odds of ANC4 utilisation were two times higher in the least poor quintile (wealthier) compared with the poorest wealth quintile [OR = 2.05; 95% CI 1.60–2.65; P = < 0.0001]. Not delivering in a health facility was associated with lower odds of ANC4 utilisation 0.54 [95% CI 0.48–0 .61; P = < 0.0001]. Lower levels of maternal education were associated with lower rates of ANC4 utilisation (Table 2). Women who got married after 18 years were more likely to utilise ANC4, but this effect was not significant [OR = 1.07; 95% CI 0.97–1.18; *P* = 0.199] while women who were married were more likely to utilise ANC4. Finally, women with children of a higher birth order (fifth or higher) were less likely to utilise ANC4 [OR = 0.87; 95% CI 0.78–0.99; *P* = 0.027] (Table 2).

**Fig. 3:**
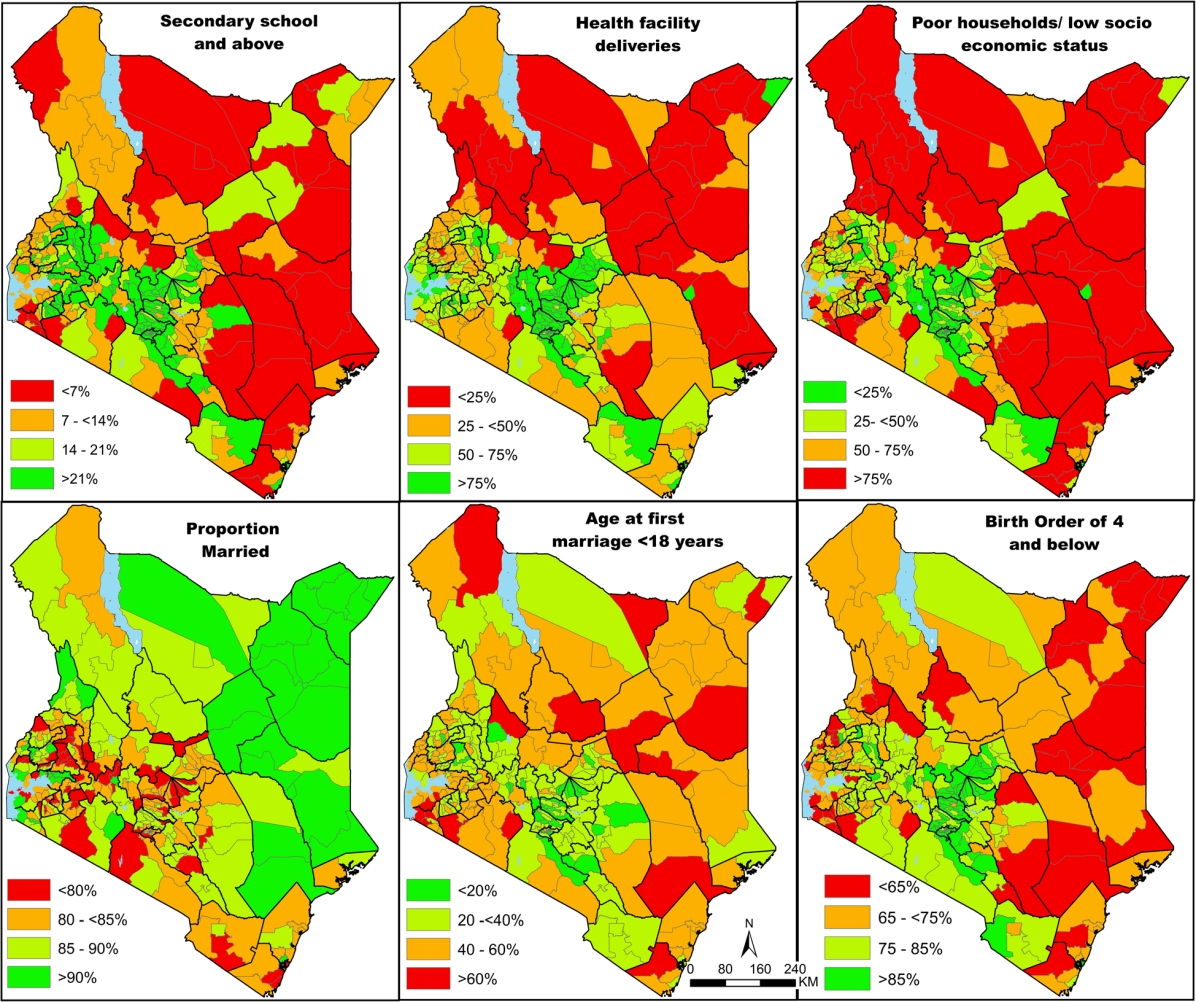
Map showing the coverage of determinants associated with the utilisation of at least 4 ANC visits at sub-county level based on the 2014 Kenya, Demographic and Health Survey from the parsimonious model. The dark lines represent the counties. Source: author generated map

Figure 3 shows the geographic variation of six determinants associated with ANC4 use in the parsimonious model in Kenya by sub-county. The spatial variation in maternal education mirrored that of the ANC4 attendance where sub-counties in central and western Kenya had higher proportions of mothers with at least secondary school education and higher coverage of ANC4 visits. Women with tertiary education were three times more likely to utilise ANC4 compared to those without any education (Fig. 3 and Table 2).

Across sub-counties, lower coverage of health facility deliveries (less than 25%) was more common in the northern, eastern and south-east areas of Kenya. In the same regions ANC4 utilisation rates were less than 50%. Similar relationships and observations were made for all the other determinants (age at first marriage, birth order and household wealth) except for marital status. For example, across sub-counties where socioeconomic status was low (> 75% of the households in the poor and poorest wealth quintiles), ANC utilisation rates were low (< 50%) (Fig. 3).

## Discussion

Improving ANC coverage across all countries is a collective priority for the global health community. Maternal mortality remains an unconscionable burden hence ensuring that maternal services reach all women equitably, including those in the poorest and most disadvantaged communities, remains a critical goal. In Kenya there has been considerable progress made towards improving ANC coverage, yet significant differences persist between sub-regions coinciding with variations in social demographic factors. ANC4 utilisation rates are heterogeneous with sub-counties in northern and eastern Kenya incredibly marginalised compared to those around central Kenya. For example, pregnant women in Central Kenya were almost five times more likely to attend the recommended four ANC visits compared to those in northern and eastern Kenya.

Regions that were disadvantaged with respect to access to ANC services bore several other disadvantages; hence geography is a critical determinant of health inequities. These areas have a higher proportion of households classified as poor, in addition to having a higher percentage of uneducated women compared to the rest of the country. Increased education levels are associated with greater use of health services, financial advantages, and greater autonomy [54–58]. Finally, these areas also had the least number of health facility deliveries highly correlated with ANC4 coverage likely due to poor road infrastructure in these areas linked to poorer metrics of geographic health access [22–24, 59–61].

ANC4 utilisation was significantly associated with one’s socioeconomic status, where women from households with high socioeconomic status were more likely to utilise a minimum of four ANC services. Socioeconomic status is strongly correlated with education where educated mothers are more aware of their health and the development of their families and have greater autonomy in deciding to use health services [62]. Women from higher social-economic groupings are also more likely to afford to seek care hence a strong predictor of higher ANC4 utilisation even in a context like Kenya where maternal services are free or highly subsidised [30–34].

The government of Kenya and other stakeholders have over the years introduced programs to improve uptake of maternal health and reduce disparities and inequities across Kenya. In June 2013, the government abolished fees payable by mothers seeking care in public health facilities, which increased health facility deliveries from 44% in 2012/13 to 62% in 2014 [34]. Under this programme (*Linda mama*), a pregnant woman is entitled to ANC, delivery, post-natal care (PNC), emergency referrals and care for infants up to one year [63]. Before the implementation of this programme, the government had a reproductive health voucher programme that was implemented between 2006 and 2016 [30–32]. The vouchers were sold at a highly subsidised price and catered for ANC, facility delivery and PNC and were specifically targeted to poor women and were associated with an increase in facility deliveries [31]. However, these subsidies didn’t appear to increase ANC coverage [31]. They resulted in a modest increase in the facility delivery and greater use of private sector for all services, further highlighting the need for interventions that are a better fit to solve the factors influencing low ANC utilisation.

The odds of having at least four ANC visits during pregnancy was significantly lower among women who were not married. Studies have shown that both economic status and dynamics regarding the distribution of power influence the use of maternal health services [64]. High birth order was also associated with a lower likelihood of utilising ANC4. There are a combination of factors likely at play here: one is the lack of time given other childcare responsibilities [65] two, is the belief among these mothers regarding their knowledge of the risks associated with pregnancy given their prior history with other pregnancies [66]. Findings such as these can guide local community-based initiatives aimed at increasing the utilisation of ANC services.

The Beyond Zero initiative launched in 2014 was aimed at complementing government programs to reduce maternal, newborn and children deaths. It focuses on promoting access to quality maternal and neonatal healthcare services and having certified centres of excellence for maternal and child health care within each county, among other priorities [67]. In addition to this, the government has set aside initiatives to improve maternal and overall health by introducing the last mile project that focusses on the establishment of health facilities to reduce travel time and influence the utilisation of interventions. Women are acutely affected by the physical and time barriers to accessing health services; however, in this study, travel time was only significant in the univariate model and its inclusion in the multivariate analysis did not improve the model fit. Kenya has a substantially high number of health facilities [21–24]. Over 98% of women who had at least one pregnancy in the five years preceding the KDHS 2014 survey lived within 30 min of the nearest health facility. Initiatives that involve the use of Community Health Workers (CHWs) are pivotal in the improvement of access to care and addressing the human resource challenges [68]. There is adequate evidence to show that CHWs have robustly improved health outcomes [68] hence the renewed attention for the need to strengthen CHWs performance. Such initiatives need subnational data to inform better targeting at levels below the county. Specifically, in marginalised sub-counties, where populations can be highly mobile, alternative, complementary approaches to existing mechanisms should be explored.

Identifying sub-counties where ANC utilisation rates remain lower and factors associated with observed patterns will allow county governments to direct suitable interventions and actions [12] to promote ANC attendance. The realisation of targets to reduce maternal mortality requires robust progress monitoring to underpin plans for improvement in health service and to identify disadvantaged groups focused on prioritising those with the greatest need. Most Government policy directives tend to be broad and frequently focus on a subset of local governing units, often failing to identify strategies that can overcome the social barriers faced by disadvantaged communities. The utilisation of insights from the existing data should be impactful in the policy formulation process and the allocation of resources to address the disparities in ANC intervention uptake. To further improve attitude and perception towards ANC, preventive and promotional health education campaigns needs to be carried out to enhance maternal health utilisation. Challenges involving adequacy of infrastructure, human resource availability and other aspects of health services provisions such as quality of care should be addressed to improve use. Besides, local governments need to utilise opportunities to leverage other non-health pro-equity interventions to increase coverage.

### Limitations

Despite the strengths of the study, there are several caveats attached to this analysis. The survey included experiences of mothers with a live birth five years preceding a survey leaving out mothers with other birth outcomes or those who might have died during pregnancy or delivery resulting in selection bias. Due to the retrospective nature of the collected data, there is a risk of recall bias which might potentially lead to inaccurate results [69]. The study was limited to the socioeconomic and demographic factors collected during the household surveys leaving out factors like availability, cost of care and skilled health workers. The displacement of cluster coordinates for confidentiality was not taken into account. Thus, a small proportion of clusters near the boundary edges may have been misclassified. However, the use of SAE models to smooth the estimates across adjacent units would potentially abate this effect.

Household sample surveys provide an opportunity to monitor the coverage and trends of most health indicators at the community level. However, these surveys are conducted every three to five years limiting tracking of trends at a higher temporal granularity. An alternative source of information is the Kenya health management Information system (HMIS) based on the District health information system Version 2 (DHIS2) which also offers information to monitor ANC trends. DHIS2 has been used to track trends and compare against those reported in the household sample surveys and is promising [70–73]. However, its use is limited due to poor reporting rates [22] and challenges in determining accurate catchment populations (population in need of service) [74].

## Conclusion

In conclusion, the ANC4 utilisation rates remain suboptimal and show substantial subnational variability. The areas with low ANC4 utilisation rates corresponded to areas of low socioeconomic status, fewer educated women and a lower number of health facility deliveries. Improvements in maternal health cannot be realised without fundamental changes in education, household wealth status, employment, and empowerment. There is need to recognise the importance of these social determinants of health as a critical driving force behind the country’s challenges with reaching targets in the health agenda related to maternal health, hence the government and stakeholders need to direct complementary measures that address social inequities.

## Supplementary information

**Additional file 1.** List of Counties (bold) and their respective sub-county (numbered) as presented in Fig. 1 of the main manuscript.

**Additional file 2.** The analytical process used to estimate the coverage ANC4 and its significant determinants at sub-county level using the 2014 Kenya Demographic and Health Survey. The datasets and outputs are shown in green while processes are shown in orange.

**Additional file 3.** The mean coverage of ANC4 in 2014 for each of the 295 sub-counties of Kenya.

## Data Availability

The full database of sample household survey (Kenya Demographic and Health Survey 2014) that supports the findings of this study is available open access from DHS program data portal- http://dhsprogram.com/data/available-datasets.cfm [7] available to registered users. The travel time surfaces are open access at 10.6084/m9.figshare.7160363 linked to work on national and sub-national variation in patterns of febrile case management in sub-Saharan Africa [37].

## Abbreviations

ANC: Antenatal care
ANC1: Proportion of women aged 15–49 years who received ANC services provided by a skilled birth attendant (doctor, nurse or midwife) at least once during pregnancy
ANC4: Proportion of women aged 15–49 years who received ANC services provided by a skilled birth attendant at least four times during pregnancy
BYM: Besag-York -Molliè
CHWs: Community health workers
KDHS2014: The 2014 Kenya Demographic and Health Survey
DHIS2: District health information system version 2
HMIS: Kenya health management Information system
MDGs: Millennium Development Goals
SSA: Sub-Saharan Africa
SDGs: Sustainable Development Goals
WHO: World Health Organization
