# Supplementary material for: Determinants of subnational disparities in antenatal care utilisation: a spatial analysis of demographic and health survey data in Kenya
Source: BMC Health Serv Res. 2020 Jul 18;20:665. doi: 10.1186/s12913-020-05531-9 (PMC7368739; doi:10.1186/s12913-020-05531-9)
Supplement: Supplementary file 1 — Additional file 1. List of Counties (bold) and their respective sub-county (numbered) as presented in Fig. 1 of the main manuscript. [file 12913_2020_5531_MOESM1_ESM.docx]

**Additional file 1: List of Counties (bold) and their respective sub county (numbered) as presented in Figure 1 of the main manuscript**

1. **Baringo county**: Baringo Central [1], Baringo North [2], Baringo South [3], Eldama Ravine [4], Mogotio [5], Tiaty [6]
2. **Bomet county**: Bomet Central [7], Bomet East [8], Chepalungu [9], Konoin [10], Sotik [11]
3. **Bungoma county**: Bumula [12], Kabuchai [13], Kanduyi [14], Kimilili [15], Mt Elgon [16], Sirisia [17], Tongaren [18], Webuye East [19], Webuye West [20]
4. **Busia** **county**: Budalangi [21], Butula [22], Funyula [23], Matayos [24], Nambale [25], Teso North [26], Teso South [27]
5. **Elgeyo Marakwet county**: Keiyo North [28], Keiyo South [29], Marakwet East [30], Marakwet West [31]
6. **Embu county**: Manyatta [32], Mbeere North [33], Mbeere South [34], Runyenjes [35]
7. **Garissa**: Balambala [36], Dadaab [37], Dujis [38], Fafi [39], Ijara [40], Lagdera [41]
8. **Homa Bay county:** Homa Bay [42], Kabondo Kasipul [43], Karachuonyo [44], Kasipul [45], Mbita [46], Ndhiwa [47], Rangwe [48], Suba [49],
9. **Isiolo county**: Garbatulla [50], Isiolo [51], Merti [52]
10. **Kajiado county**: Kajiado Central [53], Kajiado East [54], Kajiado North [55], Kajiado South [56], Kajiado West [57]
11. **Kakamega county**: Butere [58], Ikolomani [59], Khwisero [60], Likuyani [61], Lugari [62], Lurambi [63], Malava [64], Matungu [65], Mumias East [66], Mumias West [67], Navakholo [68], Shinyalu [69]
12. **Kericho county**: Buret [70], Kericho East [71], Kericho West [72], Kipkelion East [73], Kipkelion West [74], Sigowet [75]
13. **Kiambu county**: Gatundu North [76], Gatundu South [77], Githunguri [78], Juja [79], Kabete [80], Kiambaa [81], Kiambu [82], Kikuyu [83], Lari [84], Limuru [85], Ruiru [86], Thika Town [87]
14. **Kilifi county**: Ganze [88], Kaloleni [89], Kilifi [90], Magarini [91], Malindi [92], Rabai [93],
15. **Kirinyaga county**: Gichugu [94], Kirinyaga Central [95], Mwea [96], Ndia [97]
16. **Kisii county**: Bobasi [98], Bomachoge Borabu [99], Bomachoge Chache [100], Bonchari [101], Kitutu Chache North [102], Kitutu Chache South [103], Nyaribari Chache [104], Nyaribari Masaba [105], South Mugirango [106]
17. **Kisumu county**: Kisumu Central [107], Kisumu East [108], Kisumu West [109], Muhoroni [110], Nyakach [111], Nyando [112], Seme [113]
18. **Kitui county**: Kitui Central [114], Kitui East [115], Kitui Rural [116], Kitui South [117], Kitui West [118], Mwingi East [119], Mwingi North [120], Mwingi West [121]
19. **Kwale county**: Kinango [122], Lunga Lunga [123], Matuga [124], Msambweni [125]
20. **Laikipia county**: Laikipia East [126], Laikipia North [127], Laikipia West [128]
21. **Lamu county:** Lamu East [129], Lamu West [130]
22. **Machakos county**: Kangundo [131], Kathiani [132], Machakos Town [133], Masinga [134], Matungulu [135], Mavoko [136], Mwala [137], Yatta [138]
23. **Makueni county**: Kaiti [139], Kibwezi East [140], Kibwezi West [141], Kilome [142], Makueni [143], Mbooni [144]
24. **Mandera county**: Banissa [145], Lafey [146], Mandera East [147], Mandera North [148], Mandera South [149], Mandera West [150]
25. **Marsabit county**: Laisamis [151], Moyale [152], North Horr [153], Saku [154],
26. **Meru county**: Buuri [155], Central Imenti [156], Igembe Central [157], Igembe North [158], Igembe South [159], North Imenti [160], South Imenti [161], Tigania East [162], Tigania West [163]
27. **Migori county**: Awendo [164], Kuria East [165], Kuria West [166], Nyatike [167], Rongo [168], Suna East [169], Suna West [170], Uriri [171]
28. **Mombasa county**: Changamwe [172], Jomvu [173], Kisauni [174], Likoni [175], Mvita [176], Nyali [177]
29. **Murang'a county**: Gatanga [178], Kahuro [179], Kandara [180], Kangema [181], Kigumo [182], Kiharu [183], Mathioya [184], Murang'a South [185]
30. **Nairobi county**: Dagoretti North [186], Dagoretti South [187], Embakasi Central [188], Embakasi East [189], Embakasi North [190], Embakasi South [191], Embakasi West [192], Kamukunji [193], Kasarani [194], Kibra [195], Langata [196], Makadara [197], Mathare [198], Roysambu [199], Ruaraka [200], Starehe [201], Westlands [202]
31. **Nakuru county**: Bahati [203], Gilgil [204], Kuresoi North [205], Kuresoi South [206], Molo [207], Naivasha [208], Nakuru Town East [209], Nakuru Town West [210], Njoro [211], Rongai [212], Subukia [213]
32. **Nandi county**: Aldai [214], Chesumei [215], Emgwen [216], Mosop [217], Nandi Hills [218], Tinderet [219]
33. **Narok county**: Emurua Dikirr [220], Kilgoris [221], Narok East [222], Narok North [223], Narok South [224], Narok West [225],
34. **Nyamira county**: Borabu [226], Manga [227], Masaba North [228], Nyamira [229], Nyamira North [230]
35. **Nyandarua county**: Kinangop [231], Kipipiri [232], Ndaragwa [233], Ol Jorok [234], Ol Kalou [235]
36. **Nyeri county**: Kieni East [236], Kieni West [237], Mathira East [238], Mathira West [239], Mukurweini [240], Nyeri Town [241], Othaya [242], Tetu [243]
37. **Samburu county**: Samburu Central [244], Samburu East [245], Samburu North [246],
38. **Siaya county:** Alego Usonga [247], Bondo [248], Gem [249], Rarieda [250], Ugenya [251], Ugunja [252]
39. **Taita Taveta county**: Mwatate [253], Taveta [254], Voi [255], Wundanyi [256]
40. **Tana River county**: Bura [257], Galole [258], Garsen [259]
41. **Tharaka Nithi county**: Maara [260], Meru South [261], Tharaka North [262], Tharaka South [263]
42. **Trans Nzoia county**: Cherangany [264], Endebess [265], Kiminini [266], Kwanza [267], Saboti [268],
43. **Turkana county**: Loima [269], Turkana Central [270], Turkana East [271], Turkana North [272], Turkana South [273], Turkana West [274],
44. **Uasin Gishu county**: Ainabkoi [275], Kapseret [276], Kesses [277], Moiben [278], Soy [279], Turbo [280]
45. **Vihiga county**: Emuhaya [281], Hamisi [282], Luanda [283], Sabatia [284], Vihiga [285],
46. **Wajir county**: Eldas [286], Tarbaj [287], Wajir East [288], Wajir North [289], Wajir South [290], Wajir West [291]
47. **West Pokot county**: North Pokot [292], Pokot Central [293], Pokot South [294], West Pokot [295].
