# Supplementary material for: Determinants of subnational disparities in antenatal care utilisation: a spatial analysis of demographic and health survey data in Kenya
Source: BMC Health Serv Res. 2020 Jul 18;20:665. doi: 10.1186/s12913-020-05531-9 (PMC7368739; doi:10.1186/s12913-020-05531-9)

**Additional file 2:**

**The analytical process used to estimate the coverage ANC4 and its significant determinants at sub county level using the 2014 Kenya Demographic and Health Survey. The datasets and outputs are shown in green while processes are shown in orange.**


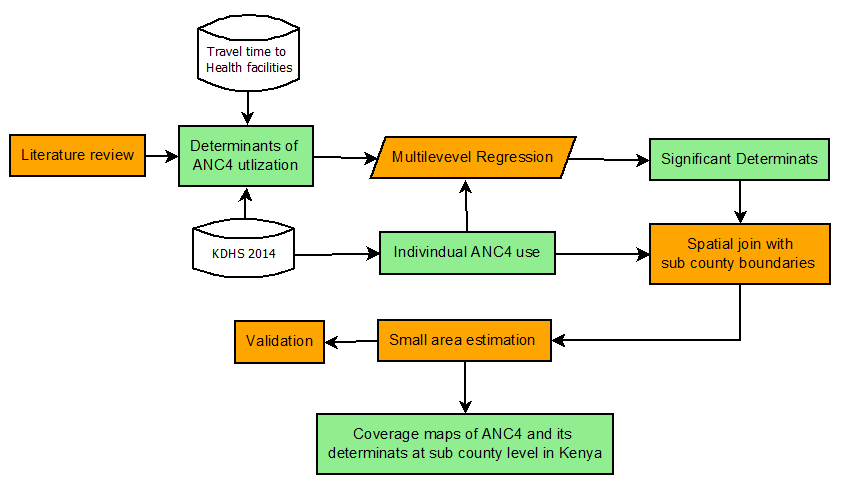

Supplement: Supplementary file 2 — Additional file 2. The analytical process used to estimate the coverage ANC4 and its significant determinants at sub-county level using the 2014 Kenya Demographic and Health Survey. The datasets and outputs are shown in green while processes are shown in orange. [file 12913_2020_5531_MOESM2_ESM.docx]
